# Spatial distribution of anti-*Toxoplasma gondii* antibody-positive wild boars in Gifu Prefecture, Japan

**DOI:** 10.1038/s41598-021-96758-x

**Published:** 2021-08-26

**Authors:** Taizo Saito, Yuko Kitamura, Eiji Tanaka, Itsuki Ishigami, Yuji Taniguchi, Junji Moribe, Katsuya Kitoh, Yasuhiro Takashima

**Affiliations:** 1 Department of Clinical Veterinary Science, The United Graduate School of Veterinary Sciences, Gifu University, 1-1 Yanagido, Gifu, 501-1193 Japan; 2 Gifu Prefectural Chuo Livestock Hygiene Service Center, 1-1 Yanagido, Gifu, 501-1112 Japan; 3 Department of Applied Veterinary Science, The United Graduate School of Veterinary Sciences, Gifu University, 1-1 Yanagido, Gifu, 501-1193 Japan; 4 Department of Veterinary Parasitology, Gifu University, 1-1 Yanagido, Gifu, 501-1193 Japan; 5 School of Social System Management, Gifu University, 1-1 Yanagido, Gifu, 501-1193 Japan; 6 Laboratory of Wildlife Resources, Gifu University, 1-1 Yanagido, Gifu, 501-1193 Japan

**Keywords:** Ecology, Microbiology

## Abstract

*Toxoplasma gondii* is a globally wide-spread parasite that infects almost all species of mammals and birds, including humans. We studied the spatial distribution of individual *T. gondii*-seropositive wild boar in Gifu Prefecture (10,621 km^2^), Japan. Altogether, 744 wild boars were captured at 663 points around human settlements in Gifu Prefecture. Serum samples were collected after recording the exact capture locations, along with each wild boar’s body length and sex. We then used a commercial enzyme-linked immunosorbent assay kit for swine to measure anti-*T. gondii* antibodies in these animals. Among the 744 wild boars, 169 tested positive for *T. gondii* (22.7%). No significant difference in *T. gondii* seroprevalence was observed between the mountainous northern region with high winter snow cover and the mild-wintered geographical plain of the southern part of the prefecture. In contrast, 8 of the 11 wild boars that were captured in a public park surrounded by residential areas showed *T. gondii* seropositivity (72.7%), a value significantly higher than those of the wild boar populations in the other prefecture areas. This in-depth analysis, which spans the big city suburbs and rural areas of a whole prefecture, explains the seroprevalence of zoonotic *T. gondii* in wild boar and has public health implications.

## Introduction

*Toxoplasma gondii*, an obligate intracellular protozoan with a worldwide distribution, causes a zoonotic disease called toxoplasmosis^1^. Toxoplasmosis can cause abortions and fetal abnormalities in pregnant woman, as well as encephalitis and brain abscesses in the immunocompromised^1^. The definitive hosts for *T. gondii* are cats and other Felidae species, and these animals shed oocysts in their feces^2^. Because the excreted oocysts persist for a long time in the soil, they can infect almost all avian and mammal species and, as one of the intermediate hosts, humans^2^. Infected intermediate hosts may harbor dormant but viable tissue cysts for years, and predators (including humans) may become infected by ingesting raw or undercooked meat contaminated with such cysts^1^. The major route for human infections is through oocyst ingestion, and exposure to soil contaminated with oocysts is one of the main risk factors for human infections^3–7^. Although the overall oocyst transmission rate for infection cases is unknown, Hill et al. suggested that about 60% of infections are oocyst-derived^8^. Therefore, understanding the status of oocyst soil contamination is crucial for better infection control.

As omnivorous animals, wild boars (*Sus scrofa*) have a strong preference for plant foods, and a meta-analysis showed that, quantitatively, more than 90% of their diet comprises plant matter^9^. Wild boars eat not only the aerial parts of plants, such as fruits, grains and seeds, but also the underground parts, such as bulbs and roots^9^. According to Kodera et al., almost all wild boars feed on the underground parts throughout the year, and in winter about 70% of the stomach contents are from underground plant material^10^. Because wild boars dig up the soil to obtain the plant parts found underground^9^, they likely have many opportunities to ingest soil-located oocysts. Although wild boars also feed on small mammals^9^ and can be infected via latent cysts in the tissues of small animals. However, habitat of such small mammals is not so wide^11–14^ and it is unlikely that these small animals have brought *T. gondii* from a distance. Therefore, wild boar infection reflects the *T. gondii* infiltration situation within each habitat of wild boar.

The seroprevalence of *T. gondii* among wild boar has been examined across the world and various infection rates, from less than a few percent to tens of percent, have been reported in different countries and regions^15^. In a meta-analysis study, Rostami et al. found that the regional pooled seroprevalence for *T. gondii* was 32.0% in North America, 26% in Europe, and 13% in Asia based on a large amount of regional information^15^. However, previous regional studies have rarely provided detailed location and density information for the animal trapping points. These data provide information on the seroprevalence of the wild boar population distributed throughout the study area, but without spatial distribution. Without information of spatial distribution, it is not possible to compare seroprevalences among subgroups based on the geographic background of the wild boar population or to verify the presence of *T. gondii* highly-contaminated “hot spots”.

In this study, we examined *T. gondii* seroprevalence among the 744 wild boars captured in 663 trapping points. Our results reveal the spatial distribution of *T. gondii* from the suburbs of big cities to the rural areas of Gifu Prefecture, spanning an area of 10,621 km^2^.

## Results

### Setting the cut off value for ELISA

**Figure 1:**
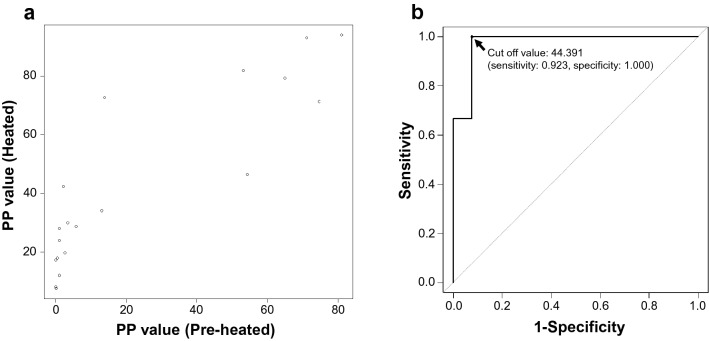
Relationship between the Percentage of Positivity (PP) values from the pre- and post-heated serum samples measured with the ELISA system to detect anti-*T. gondii* antibodies. (**a**) PP values of the 19 sera from pigs experimentally infected with *T. gondii*. Each circle represents the data for each wild boar. (**b**) ROC curve used for calculating the cut-off value for the post-heated samples. The curve was created on the assumption that the results from the pre-heated samples are correct.

In this study, we examined *T. gondii* seroprevalence using wild boar serum library belonging a department of livestock hygiene in Gifu prefecture administration. The administration provided the serum library after heat-inactivation to prevent the spread of pathogens that may be contained. Although commercially provided ELISA kit, PrioCHECK Toxoplasma Ab porcine ELISA kit (Thermo Fisher Scientific Prionics, Carlsbad, CA), to detect anti-*T. gondii* antibodies used in this study does not mention the need to avoid heat-treatment of the specimen serum, it has been reported that heat treatment of serum may affect the results of some ELISA systems^16^. Therefore, we measured the OD value of 19 pig serum samples before and after heat-treatment (56 °C for 30 min, 65 °C for 30 min) using the commercially provided ELISA kit. And the PP value, numerical value obtained by correcting inter-plate error based from OD value, were calculated according to manufacturer’s instructions. The aforementioned pigs had been previously experimentally infected with *T. gondii* and their sera showed various antibody titers before the heat-treatment^17^. As a result, an increased absorbance was observed in some samples (Fig. 1a). Hence, a new cut-off value for positive judgment was set for the heat-treated serum samples as follows. First, the positive judgement used for the pre-heat-treated samples based on the manufacturer’s instructions was considered to be a true positive result. Next, a receiver operating characteristic (ROC) curve was created using the PP value for the heat-treated samples as described below (Fig. 1b). Based on this curve, a cut-off value for the heat-treated samples was set (PP > 44.391). The sensitivity and specificity were 0.923 and 1.000, respectively.

### Prevalence of anti-*T. gondii* antibodies among wild boars in Gifu Prefecture, Japan

Blood samples from 744 of these animals at 663 points in Gifu Prefecture (10,621 km^2^), Japan were tested by ELISA. These serum samples were previously obtained from 319 piglets (42.9%) and 425 adults (57.1%), and from 393 males (52.8%) and 351 females (47.2%) (Table 1). Altogether, 169/744 boars (22.7%, 95% CI: 19.8–25.9%) showed *T. gondii* seropositivity. Seropositivity differed significantly between piglets and adults (p = 2.617 × 10^–5^, Fisher’s exact test) but not between males and females (p = 0.6617, Fisher’s exact test).

**Table 1:** Fisher's exact test for anti-*T. gondii* antibody seropositivity in wild boars.

			Positive no. (%)	Negative no. (%)	p-value
Total		744	169 (22.7)	575 (77.3)	
Growth	Piglet	319	43 (13.5)	276 (86.5)	p = 2.617 × 10^–5^
	Adult	425	126 (29.6)	299 (70.4)	
Sex	Male	393	92 (23.4)	301 (76.6)	p = 0.6617
	Female	351	77 (21.9)	274 (78.1)	
Area	Hida	71	12 (16.9)	59 (83.1)	p = 0.3752
	Mino	673	157 (23.3)	516 (76.7)	
	Area shown in Fig. 2c	11	8 (72.7)	3 (27.3)	p = 5.373 × 10^–4^
	Other areas	733	161 (22.0)	572 (78.0)	

**Figure 2:**
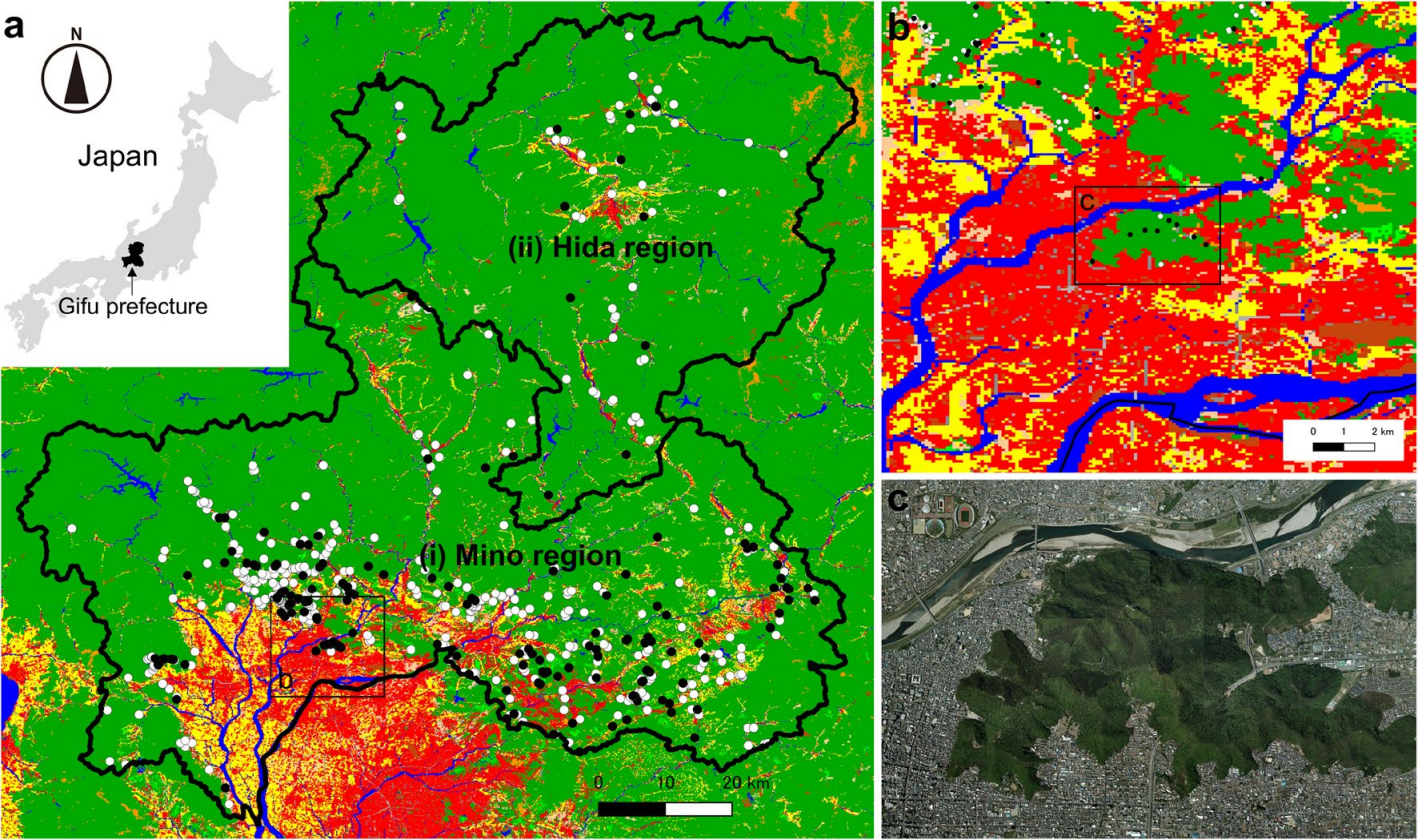
Spatial distribution of anti-*T. gondii* antibody-positive wild boars in Gifu Prefecture, Japan. (**a**) Locations of the capture points of the examined wild boars. Black and white filled circles represent each capture point for the seropositive and negative wild boars, respectively. The colors on the map indicate the land use status as follows. Green: forests, Red: residential areas, Yellow: rice fields, pale orange: other agricultural land, Orange: wastelands, Blue: rivers and lakes, Light green: golf courses. Among the 663 capture points, multiple wild boars were captured at 68 points. When all the wild boars captured at a point were *T. gondii*-negative, a white filled circle is shown. A black filled circle is shown even when a single animal was *T. gondii*-positive. Figure was created with Quantum Geographic Information System (QGIS) software (version 3.12.3-București)^34^. (**b**) Enlarged view of the area surrounded by a square in panel (**a**). Residential areas surrounding the park. (**c**) Aerial photograph of the area shown in panel (**b**). The image was downloaded from the Map and Aerial Photograph Browsing Service (https://mapps.gsi.go.jp/maplibSearch.do#1) of the Geospatial Information Authority of Japan (GSI). These data were partially processed and used.

Gifu Prefecture can be divided into two regions: (i) Mino region and (ii) Hida region (Fig. 2a). Mino region is close to the metropolis and has a high population density (Fig. 2a and Supplementary Table S1). Most of the Mino region is low altitude with little annual snowfall (Supplementary Table S1), whereas Hida is far from the metropolis, has a low population density, and is a mountainous region with high annual snowfall (Fig. 2a, Supplementary Table S1). However, despite their different environments, a significant seroprevalence difference was not observed between Mino and Hida regions (p = 0.3752, Fisher’s exact test) (Table 1).

The wild boar population in Gifu Prefecture was reported to be divided into four subgroups based on genetic and geographical background^18^ (Fig. S1). The seroprevalence of each subgroup was shown in Table S2. There was no significant difference in seroprevalence between the groups (Table S3).

Although most wild boars were captured in the forest area, or in the boundary between the residential and forest areas (Fig. 2a), 11 were captured in the park of a large urban area in the southern part of the prefecture (Fig. 2b,c). The park’s area is approximately 5.92 km^2^ and is surrounded by the residential area of Gifu city in which the population is approximately 400,000. In this park, the boars rarely leave the area and boars from outside rarely enter it ^19^. In this almost isolated environment, 11 samples were obtained and eight sera showed seropositivity (72.7%, 95% CI: 39.0–94.0%) (Fig. 2c), a seroprevalence significantly higher than that of the other areas (p = 5.373 × 10^–4^, Fisher’s exact test) (Table 1).

### Ignorable levels of cross-reactivity with anti*-Sarcocystis* antibodies

**Figure 3:**
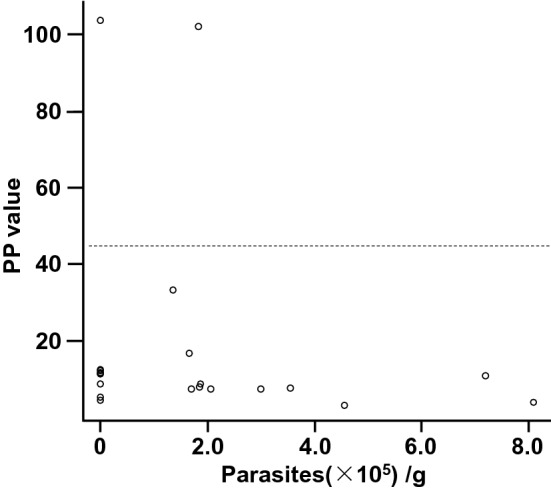
Cross-reactivity of antibodies induced by *Sarcocystis* infection was negligible when using ELISA kit designed for anti-*T. gondii* antibodies. Correlation between the number of bradyzoites in the diaphragm and the PP values of the commercial ELISA system used in this study. Each dot represents data from each wild boar. The dotted line indicates the cut-off line of the ELISA system.

The observed high seroprevalence (22.7%) prompted us to confirm the possibility of cross-reactive anti-*Sarcocystis* antibodies in the ELISA system we used to detect anti-*T. gondii* antibodies, because it has been reported that dozens of wild boars in the Gifu Prefecture are infected with *Sarcocystis* spp.^20^ *Sarcocystis* are protozoans closely related to *T. gondii*^21^, and cross-reactivity between anti-*T. gondii* antibodies and anti-*Sarcocystis* spp. antibodies have been reported^22^. To this end, we evaluated the relationship between the number of *Sarcocystis* spp. bradyzoites in the diaphragm tissue (Fig. S2) and serum anti-*T. gondii* antibody levels (as measured by the commercially provided ELISA system) in the 21 wild boars. As a result, *Sarcocystis* spp. were found in 12/21 (57.1%) of the diaphragm specimens, but of the 12 *Sarcocystis*-positive wild boars, 11 were negative by ELISA (Fig. 3). These results clearly indicate that *Sarcocystis* infection did not affect the specificity of the ELISA system we used, regardless of the high *Sarcocystis* infection rate in the examined areas and the potential immune crossover between *T. gondii* and *Sarcocystis* spp.

## Discussion

In this study of more than 700 serum samples, we determined the spatial distribution of *T. gondii*-infected wild boars in Gifu Prefecture (10,621 km^2^), Japan. Many studies have reported on the seroprevalence of *T. gondii* among wild boars in several areas^15^, but data has been missing on the number of samples and/or the information of the capture points, and the detailed spatial distribution patterns in the examined areas were not described. We overcame this problem by secondary usage of the large numbers of serum samples previously collected by the Prefectural Government. Thus, this study provides a good example of the effective reuse of large numbers of specimens previously collected by a government agency.

Between Hida and Mino regions, of which human population density and climate are different, no statistical difference of seroprevalences was observed. Four wild boar subgroups with different genetic and geographical backgrounds also showed similar seroprevalence. On the other hand, the seroprevalence of a wild boar in the isolated park in an urban area, was significantly higher than that of other population. These results suggest that there are local “hot spots” of *T. gondii* contamination but *T. gondii* parasites widely distribute all over the Gifu prefecture regardless human populations nor climate.

Using variety test systems including the commercial ELISA kit used in this study, seroprevalence of wild boar has been investigated in many countries and regions^23,24^. Among them, Dubey et al. succeed to isolated viable *T. gondii* parasites from multiple seropositive wild boars^25^, suggesting reliability of the serological tests. In this report, seroprevalence of wild boars in USA was reported as 27.7%. The seroprevalence of 22.7% obtained in our study is of the same magnitude as in USA.

*Sarcocystis* spp. and *T. gondii* are closely related protozoans, the former being common among wild boars across the world^20,26–29^. Hence, it is not surprising that anti-*T. gondii* antibody cross-reactivity with *Sarcocystis* antigen has been reported^22^. Therefore, when wild boar sera are examined for *T. gondii* infection, careful consideration should be given to the possibility that the sera from *Sarcocystis*-infected wild boars will produce false positives. However, as far as we know, no studies have considered such cross-reactivity in the various anti-*T gondii* antibody detection systems that had been used for wild boar in the past. We have previously confirmed that numerous wild boars in Gifu Prefecture harbor latent *Sarcocystis* spp. in their muscle tissues^20^. Because the *T. gondii* positivity rate in this study was relatively close to the previously reported *Sarcocystis* infection rate, we decided to examine this cross-reactivity using a commercial ELISA system in this study. Most of the *Sarcocystis*-infected wild boar samples were judged to be negative by the commercial ELISA kit (Fig. 3). Our results show that the ELISA system we used herein was not affected antibodies induced by *Sarcocystis* infection.

In Gifu Prefecture, there are 32 industrialized pig farms as of 2019 (Gifu Prefecture administrative document; https://www.pref.gifu.lg.jp/page/12716.html). There are no households with pigs in this area. The reported seroprevalence among pigs slaughtered at abattoirs in Gifu Prefecture, 5.2% (8/115 pigs)^30^ is much lower than seroprevalence in wild boars revealed in this study. These results indicate the control of *T. gondii* infection was somewhat successful in pig farms in Gifu Prefecture.

Wild boars are omnivorous with a strong preference for plant foods, and they dig up soil and feed on plant roots and insects in this medium^9^. In addition, wild boar also eats rodents, in certain occasions carcasses of mammals^9^. These feeding habits may give wild boars the opportunity to infect both oocysts in soil and tissue cysts. The ranging behavior of wild boars is small, usually around 4 km^2^^31^. In addition, the home range of rodents, which are the main prey of wild boars, is narrow. For example, the rodents distributed in the study area, such as *Apodemus speciosus*, *Apodemus argenteuss*, and *Mus musculus*, have a home range of about 1000–3000 m^2^, and *Lepus brachyurus*(Japanese hare) has a home range of 0.12km^2^^11–14^. Thus, it is unlikely that rodents from far outside the boar's territory are being preyed upon. Therefore, it can be assumed that the seroprevalence of wild boars reflects the local environment contamination level with *T. gondii*. The distribution of seropositive wild boars throughout Gifu Prefecture suggests extensive environmental pollution presence around the human settlements in this region. Although the situation in the residential area of the city, which wild boar do not inhabit, cannot be directly assumed, wild boars captured in the park surrounding the residential area showed a high *T. gondii* seroprevalence (Fig. 2c, Table 1). This result suggests that wild boars around urban areas have a higher seropositivity rate than those around local areas. The wild boar population in this park is considered to be almost isolated^19^, so the high infection rate in this population likely reflects high-level *T. gondii* contamination in the surrounding residential area.

In Japan, about 43,000 stray cats were captured by local government agencies in 2019 (Ministry of the Environment, Japan; https://www.env.go.jp/nature/dobutsu/aigo/2_data/statistics/dog-cat.html). These cats roam freely outside and are considered the primary source of oocyst soil contamination. High-level oocyst contamination possibly occurs in the human populated urban area, where many cats also roam freely outside. Free-roaming and feral cats are now being considered alien animals in that they prey on rare local species and bring *T. gondii* infections into the habitats of wild animals^32,33^. Although such problems are noted in the nature-rich areas inhabited by rare and endemic species, little attention is paid to urban settlements. It is therefore necessary to investigate the oocyst pollution situation in residential areas but this cannot be revealed by wild boar surveys. Although direct detection of oocysts in soil lacks coverage sensitivity and is not suitable for surveys, an indirect investigation may be possible, such as antibody investigations on rodents living in urban areas. In any case, considering the extremely high *T. gondii* seroprevalence in wild boar in the urban area, promoting the lifelong habit of keeping cats indoors would help to control zoonotic transmission of toxoplasmosis.

## Methods

### Ethical statement

According to the Regulations on Animal Care and Use in Research of Gifu University, it was confirmed that this study was not considered as animal experiment which requires approval of this committee, because no wild boars in this study were killed specifically for this study. The risk of infectious diseases was managed using facilities and methods approved by the Pathogen Safety Management Committee of Gifu University (approved number: 1gidaikennkenn3gou-6). This study was carried out in compliance with the ARRIVE guidelines (https://arriveguidelines.org).

### Collection of serum samples for determine seroprevalence

In 2018, an outbreak of classical swine fever (CSF) was observed in pigs for the first time in 26 years in Japan. The infection was later also confirmed in wild boar in the Gifu Prefecture. Hence, large-scale wild boar capture surveys were conducted in Gifu Prefectural government. All the wild boars in this survey were captured by licensed hunters in accordance with the Protection and Control of Wild Birds and Mammals and Hunting Management Law. Altogether, 744 serum samples were collected from wild boar captured at 663 points in Gifu Prefecture, Japan, in accordance with Specific Domestic Animal Infectious Disease Quarantine Guidelines for CSF from the Japanese government (from September, 2018 to June, 2020). Data on capture points, length and sex were recorded for each wild boar (Supplementary Data). Animals with body lengths exceeding 90 cm were defined as adults, and those below 90 cm were defined as piglets. After CSF surveillance by the local government, the remaining serum samples were heat-treated twice (56 °C for 30 min, and 65 °C for 30 min) to inactivate the CSF virus and then used in this study.

### Map depiction

Land use data were downloaded from the Land Use Subdivision Mesh Data (Raster Version) Version 2.5, National Land Information Division, National Spatial Planning and Regional Policy Bureau, MLIT of Japan (https://nlftp.mlit.go.jp/ksj/gml/datalist/KsjTmplt-L03-b_r.html). Administrative division data were downloaded from the Administrative Zones Data (Version 3.0), National Land Information Division, National Spatial Planning and Regional Policy Bureau, MLIT of Japan (https://nlftp.mlit.go.jp/ksj/gml/datalist/KsjTmplt-N03-v3_0.html). These data were partially processed and used. Land use data, administrative division data, and sampling location data were superimposed using the Quantum Geographic Information System (QGIS) software (version 3.12.3-București)^34^.

### Verification for cross reactivity between anti-*T. gondii *and anti-*Sarcocystis* spp

Sera and the diaphragm specimens were collected from 21 wild boars captured in Gifu Prefecture before 2018 for the control of harmful mammals and meat, in accordance with the Protection and Control of Wild Birds and Mammals and Hunting Management Law. Samples were stored at − 20 °C. Individual diaphragm sample weights in this study ranged from 8.0–41.3 g. The whole samples were digested using pepsin solution, and the *Sarcocystis* spp. tissue bradyzoites were collected as previously described^35^. The number of bradyzoites obtained was counted and the density (number of bradyzoite/1.0 g of diaphragm tissue) was calculated based on the weight of each diaphragm specimen.

### Statistical analysis

Fisher’s exact test was used for the seroprevalence comparisons. ROC curve drawing was performed in the R version 4.0.2^36^ with the R-package “pROC”^37^.

## Supplementary Information

Supplementary Information.

## Data Availability

The datasets generated during the current study are available from the corresponding author on reasonable request. All data analyzed during this study are included in this published article (and its Supplementary Information files).
