# Supplementary material for: Spatial distribution of anti-Toxoplasma gondii antibody-positive wild boars in Gifu Prefecture, Japan
Source: Sci Rep. 2021 Aug 26;11:17207. doi: 10.1038/s41598-021-96758-x (PMC8390498; doi:10.1038/s41598-021-96758-x)
Supplement: Supplementary file 1 — Supplementary Information. [file 41598_2021_96758_MOESM1_ESM.pdf]

(Supplementary information)

Spatial distribution of anti-*Toxoplasma gondii* antibody-positive wild boars in Gifu Prefecture, Japan

Taizo Saito, Yuko Kitamura, Eiji Tanaka, Itsuki Ishigami, Yuji Taniguchi, Junji Moribe, Katsuya Kitoh, Yasuhiro Takashima

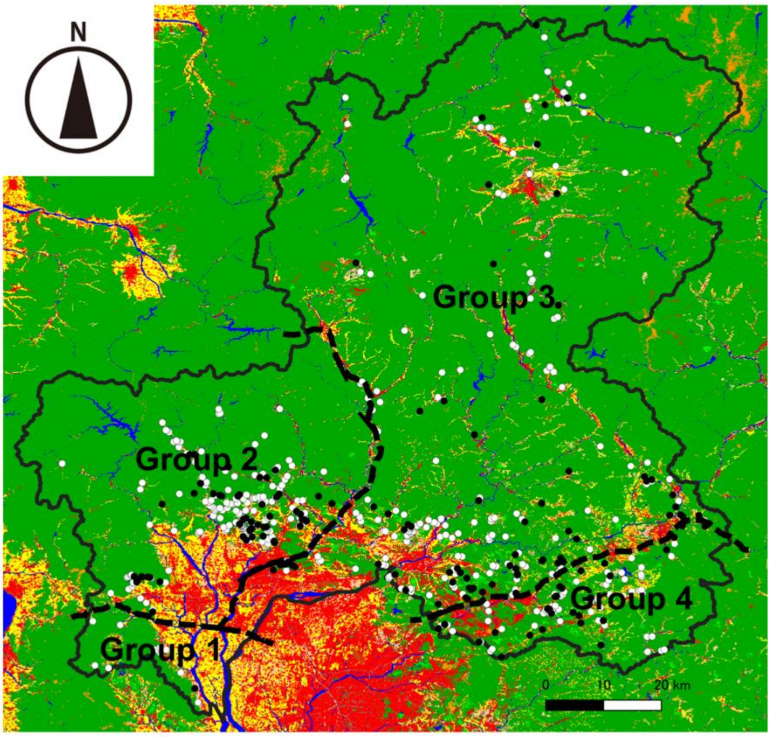

Supplementary Fig. S1 Distribution of four wild boar subgroups in Gifu Prefecture are shown. The wild boar population was divided into four major subgroups based on genetic and geographical background[18]. The colors on the map indicate the land use status as follows. Green: forests, Red: residential areas, Yellow: rice fields, pale orange: other agricultural land, Orange: wastelands, Blue: rivers and lakes, Light green: golf courses. Among the 663 capture points, multiple wild boars were captured at 68 points. When all the wild boars captured at a point were *T. gondii*-negative, a white filled circle is shown. A black filled circle is shown even when a single animal was *T. gondii*-positive. Land use data were downloaded from the Land Use Subdivision Mesh Data (Raster Version) Version 2.5, National Land Information Division, National Spatial Planning and Regional Policy Bureau, MLIT of Japan (https://nlftp.mlit.go.jp/ksj/gml/datalist/KsjTmplt-L03-b_r.html). Administrative division data were downloaded from the Administrative Zones Data (Version 3.0), National Land Information Division, National Spatial Planning and Regional Policy Bureau, MLIT of Japan (https://nlftp.mlit.go.jp/ksj/gml/datalist/KsjTmplt-N03-v3_0.html). These data were partially processed and used. Land use data, administrative division data, and sampling location data were superimposed using the Quantum Geographic Information System (QGIS) software (version 3.12.3-București, https://qgis.org/en/site/forusers/download.html)[34].

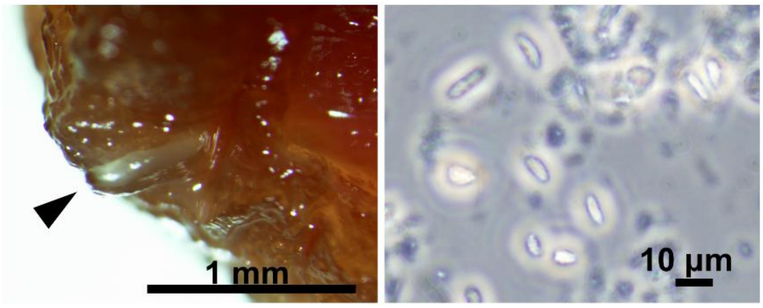

Supplementary Fig. S2 *Sarcocystis* spp. detected in the diaphragm tissue from a wild boar. Left: A sarcocyst, one of the parasite life-stage forms, with many bradyzoites shown inside. Right: Bradyzoites, small parasite bodies residing within sarcocysts.

Supplementary Table S1. Elevation and weather information for representative cities in the Mino and Hida regions

Region	City	Elevation(m)[a,1]	Population[b,2]	Temperature(°C)[c]			Snow accumulation(cm)[c]	Reference[d]
				Annual	August	January	Annual (total)	
Mino	Gifu	44.7	402,670	15.8	28.0	4.4	47	3
	Ogaki	12.8	159,095	15.8	27.9	4.5	no data	4
	Tajimi	168.8	108,061	14.8	27.4	2.9	no data	5
Hida	Hida	696.3	23,406	11.2	24.1	-0.9	601	6
	Takayama	667.7	86,854	11	24.1	-1.4	473	7
	Shirakawa	486.8	7,713	10.7	23.5	-1.1	1056	8

[a]Average elevations of the residential areas of each city.

[b]As of October, 2018.

[c]Average measurements from 1981 to 2010.

[d]References for temperature and snow accumulation.

References

1. Zaiki M., Oguchi T., Kagawa Y., Takahashi A., Koike S., Yamauchi M. Relationships between Distribution of Japanese Residential Areas and Topography, Annual Meeting of the Association of Japanese Geographers; Spring 2005. https://doi.org/10.14866/ajg.2005s.0.188.0 (2005).
2. Gifu prefecture official HP, https://www.pref.gifu.lg.jp/page/19060.html
3. Japan Meteorological Agency HP, https://www.data.jma.go.jp/obd/stats/etrn/view/nml_sfc_ym.php?prec_no=52&block_no=47632&year=&month=&day=&view=
4. Japan Meteorological Agency HP, https://www.data.jma.go.jp/obd/stats/etrn/view/nml_amd_ym.php?prec_no=52&block_no=0496&year=&month=&day=&view=
5. Japan Meteorological Agency HP, https://www.data.jma.go.jp/obd/stats/etrn/view/nml_amd_ym.php?prec_no=52&block_no=1058&year=&month=&day=&view=
6. Japan Meteorological Agency HP, https://www.data.jma.go.jp/obd/stats/etrn/view/nml_amd_ym.php?prec_no=52&block_no=0473&year=&month=&day=&view=
7. Japan Meteorological Agency HP, https://www.data.jma.go.jp/obd/stats/etrn/view/nml_sfc_ym.php?prec_no=52&block_no=47617&year=&month=&day=&view=
8. Japan Meteorological Agency HP, https://www.data.jma.go.jp/obd/stats/etrn/view/nml_amd_ym.php?prec_no=52&block_no=1306&year=&month=&day=&view=

Supplementary Table S2.

Seroprevalence of wild boars in each subgroup

		Positive No. (%)	Negative No. (%)
Group 1	12	1 (8.3)	11 (91.7)
Group 2	311	58 (18.6)	253 (81.4)
Group 3	291	75 (25.8)	216 (74.2)
Group 4	130	35 (26.9)	95 (73.1)
Total	744	169	575

Supplementary Table S3.

Pairwise comparisons using Fisher's exact test with Holm's correction

	p-value
Group1:Group2	1
Group1:Group3	1
Group1:Group4	1
Group2:Group3	0.235
Group2:Group4	0.279
Group3:Group4	1
